# Preliminary Evaluation of the Effect of Domestication on the Marketable and Nutritional Quality of *B. aegyptiaca* (L.) Delile Oil from Algeria

**DOI:** 10.3390/foods13172752

**Published:** 2024-08-29

**Authors:** Wafaa Amira Slimani, Ambrogina Albergamo, Rossana Rando, Vincenzo Nava, Mohamed Ould Safi, Sidi Mohammed Bachir Bensenane, Vincenzo Lo Turco, Benamar Benmahioul, Giuseppa Di Bella

**Affiliations:** 1 Department of Forest Resources, Faculty of Natural and Life Sciences, Earth and Universe, University of Tlemcen, Tlemcen 13000, Algeria; wafaa.amira.frt@gmail.com (W.A.S.); benmahioul@yahoo.fr (B.B.); 2 Department of Biomedical, Dental, Morphological and Functional Images Sciences (BIOMORF), University of Messina, Viale Annunziata, 98122 Messina, Italy; rrando@unime.it (R.R.); vloturco@unime.it (V.L.T.); gdibella@unime.it (G.D.B.); 3 Department of Veterinary Science (SCIVET), University of Messina, Viale Annunziata, 98168 Messin, Italy; 4 National Institute of Forest Research, Adrar Station, Adrar, Algeria; moh.safi.inrf@gmail.com; 5 Faculty of Nature and Life Sciences and Earth and Universe Sciences, Laboratory of Physiology, Physiopathology and Biochemistry of Nutrition-PPABIONUT, AbouBekr Belkaid University of Tlemcen, Tlemcen 13000, Algeria; bachirbdz@yahoo.fr

**Keywords:** desert date, domestication, edible oil, chemical composition, fatty acids, sterols, inorganic elements, tocopherols, total polyphenols, squalene

## Abstract

*Balanites aegyptiaca* is a multipurpose fruit tree that grows wild in many arid and semi-arid African areas; however, recent domestication efforts have been undertaken to protect the species from the threat of urbanization and climate change. Within this context, the impact of the domestication of Algerian *B. aegyptiaca* was evaluated on its seed oil, which is already valued as food. Hence, oils from wild and domesticated trees were comparatively investigated for their physicochemical and compositional quality. Both oil types had a good oxidative stability and met the requirements for human consumption in terms of the saponification index, the free acidity, and the peroxide value. Moreover, they showed a comparable FA composition, with high levels of oleic and linoleic acids, which are beneficial for the consumer’s health. Domestication led to a statistically significant decrease in the tocopherols and polyphenols in the oil. The phytosterols and squalene were slightly lower in the domesticated oil than in the wild relative, although no statistically significant differences were observed. A comparable mineral profile was revealed and the minimal variations in the trace elements between the oils could be related to the natural variability in the seeds. Hopefully, this study will encourage the domestication of *B. aegyptiaca* as a sustainable strategy for enhancing its socioeconomic value in Algerian rural areas.

## 1. Introduction

*Balanites aegyptiaca* (L.) Delile (family Zygophyllaceae) is a neglected fruit tree, native to a large part of Africa and to limited areas of the Middle East. The spiny tree, which stands up to 10 m tall, starts to produce fruit at 5–7 years old and reaches its maximum fruit bearing capacity in 15–25 years, with an annual production capacity of 100–150 kg of fruit per tree [1]. The fruit is a drupe (2.5–7 cm in length and 1.5–4 cm diameter) that is green and pubescent when unripe, and glabrous and yellowish after ripening. It consists of four layers: the epicarp, the fleshy mesocarp, the woody endocarp, and the seed or kernel [2]. The species adapts to many soil types (e.g., sandy, stony, clayey, and even alluvial soils), and naturally grows in plains, oases, depressions, and areas up to 1000 m above sea level. It is also considered a salinity- and drought-tolerant tree, as it lives with temperatures and rainfall varying between 20 to 30 °C and 100 to 1000 mm, respectively [3], thus making it ideal for combating desertification and restoring degraded land [4]. Due to its considerable ecological plasticity, *B. aegyptiaca* is widely distributed in arid and semi-arid African regions, but it is also present in the Middle East (Arabian Peninsula) and some regions of south Asia (e.g., Israel, Jordan, Saudi Arabia, Yemen, India, and Myanmar) [4,5]. In African rural communities, it is a key agroforestry species, with high cultural, social, and economic value, not only in terms of the abundance of fruit produced, but also in terms of the various plant organs that are used in the provision of several goods and services, thus substantially contributing to the welfare of such communities [6]. In fact, young leaves and shoots are consumed as vegetables [7]. In addition to this, adult leaves are considered as food and feed, with interesting hepatoprotective, antioxidant, and antimicrobial properties [7,8,9]. In traditional medicine, extracts and infusions of the roots and bark can heal several disease-related conditions, from syphilis, yellow fever, and epilepsy to hypertension and gastrointestinal disorders [10,11]. The wood serves not only as firewood and as a material in charcoal production, but also serves as a raw material for making furniture and various household items [12]. The fruit, commonly known as “desert date”, is the organ of the plant with the highest economic value. It is particularly appreciated as a food for its bitter-sweet taste and hypoglycemic properties [13]; it can be consumed raw, fermented to produce alcoholic beverages, or even used for preparing porridge and pancakes [5,14]. The kernel can be consumed as a nut after boiling [14], or pressed to extract up to 60% oil, which is highly valued not only in human nutrition [15,16], but also in traditional medicine [17] and in the production of biodiesel [18]. In this regard, the literature reports that studies of this oil have been mainly carried out in relation to the geographical area of origin [19,20,21,22,23] and the health-related properties [24,25].

However, the loss of natural habitats experienced in Africa since the 1990s, due to urbanization, the expansion of agricultural areas, the misuse of forest resources and, not least, climate change, has affected the indigenous distribution of *B. aegyptiaca* and will likely lead to its extinction in the next few decades [26,27]. Despite the desert date tree having been recognized as one of the candidates of African species for domestication and integration into agroforestry systems, just a few conservation and cultivation measures involving this species have been conducted to preserve its genetic diversity and valorize its multipurpose character [12,28]. In Algeria, for example, *B. aegyptiaca,* locally known as tabourak, represents a dominant indigenous species, growing naturally in the provinces of Adrar, Tamanrasset, and Illizi, and it is unfairly underutilized and undervalued compared to other areas of the Sahel region [29]. To the best of the authors’ knowledge, no attempts have yet been made to domesticate this species in this country to sustain and improve local livelihoods.

The domestication of indigenous fruit trees, traditionally important for their marketable food products, includes both cultivation and genetic improvement. The idea originated in the 1980s [30] and was subsequently taken up by the World Agroforestry Centre in 1993 [31]. Since then, it has become a global programme with a strong African focus, expanding both in terms of the number of candidate species for domestication and the range of research topics covered by its multidisciplinary approach and, to date, targeting 14 of the 17 UN Sustainable Development Goals [32].

Based on these premises, the aim of the current work is to conduct a preliminary study on the impact of domestication of *B. aegyptiaca* on the marketable and nutritional quality of its seed oil. For this purpose, the oil from wild and cultivated trees cohabiting a specific area in southwestern Algeria were investigated for their yield, as well as their physicochemical and compositional properties. Due to the socioeconomic relevance of desert date oil, this study is crucial not only for promoting the cultivation of *B. aegyptiaca* in Algeria, but also for encouraging the sustainable use of such oil in the food sector.

## 2. Materials and Methods

### 2.1. Plant Materials and Experimental Assay

**Figure 1:**
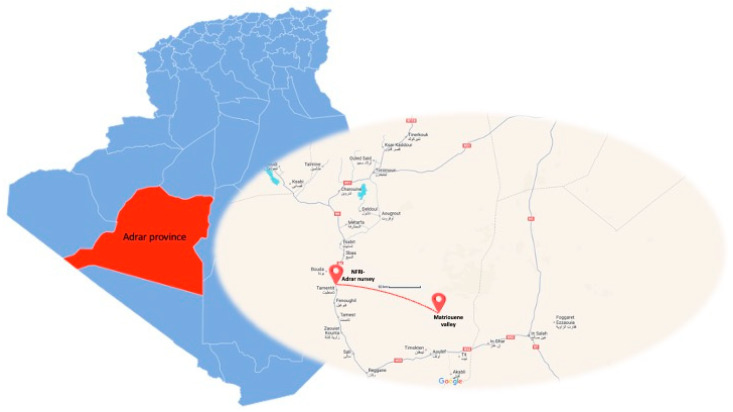
Geographical map of the provinces in Algeria. The province of Adrar is highlighted in red and an enlargement of the sampling area is shown on the left.

The plant materials in this study were collected during November 2023 and consisted of wild and domesticated *B. aegyptiaca* fruits harvested from two locations in the province of Adrar (Algeria), respectively, a valley in Matriouene and Adrar nursery, part of the National Forest Research Institute (NFRI). These areas are about 50 km away from each other and share the same geopedoclimatic context, as reported in Figure 1 and Table 1.

The domestication of *B. aegyptiaca* was carried out at an experimental plot installed in February 2014 at Adrar nursery, as already described by Slimani et al. [29]. Specifically, 63 trees were propagated from wild seeds, by employing an irrigation regime that consisted of watering the plants three times a week, without any chemical input. Periodic pruning was also carried out to encourage healthy growth and maximize fruit production.

For the fruit collection, *n* = 600 ripe and healthy fruits (~2 kg) were manually harvested both from wild (*n* = 15) and domesticated (*n* = 15) adult and productive trees, totaling *n* = 1200 fruits (~4 kg). The fruits were transported from both sampling sites to the University of Tlemcen and stored in the dark and at a constant room temperature, until oil extraction. Additionally, the collected plant materials were authenticated by a botanist from the Department of Forest Resources at the same university and the wild and domesticated voucher specimens (BE-W 035.23 and BE-D 034.23) were deposited in the department’s herbarium.

### 2.2. Oil Extraction

The oil extraction occurred, according to the cold-pressing procedure. Briefly, the fruits were carefully stripped of their pulp and the raw kernels were crushed with a metal hammer. Then, *B. aegyptiaca* oil was extracted using a stainless-steel oil press, manufactured by YASON (China). The press cage was first heated to a temperature not exceeding 50 °C, for approximately 10 min. Then, the *B. aegyptiaca* seeds were placed inside and manually pressed to extract the oil. The oil was collected through perforations in the press cage that allowed the oil to run into a container below and the yield from the extraction was gravimetrically determined. The oil samples were first centrifuged to remove particle residues and then stored in amber glass bottles, which were labelled according to their origin: “domesticated oil” from the Adrar nursery and “wild oil” from the Matriouene valley. To check the reproducibility of the oil extraction process, 3 samples of domestic oil and 3 samples of wild oil were used in the present study.

### 2.3. Reagents and Materials

The reagent-grade solvents (i.e., n-heptane, n-hexane, diethyl ether, and methanol) were provided by J.T. Baker (Phillipsburg, NJ, USA), while the HPLC-grade solvents (i.e., n-hexane, ethyl acetate, methanol, and water) were purchased from LiChrosolv (Merk, Darmstadt, Germany). Bis-trimethylsilyl-trifluoroacetamide and trimethylchlorosilane (BSTFA:TMCS 99:1) and silica gel type G, with ~13% calcium sulfate (high-purity grade), were supplied by Supelco (Bellefonte, PA, USA). The reference standards (C4–C24) for fatty acid methyl esters (FAMEs) and the analytical standards for single sterols (cholesterol, brassicasterol, 24-methylen-cholesterol, campesterol, campestanol, stigmasterol, Δ-7-campesterol, clerosterol, β-sitosterol, Δ-5-avenasterol, Δ-5,24-stigmastadienol, Δ-7-stigmastenol, and Δ-7-avenasterol, ≥98% purity each), single tocopherols (α-tocopherol, γ-tocopherol, δ-tocopherol, 98% purity each), gallic acid (99% purity), and squalene (≥98% purity) were all from Sigma-Aldrich (St. Louis, MO, USA) and Supelco (Bellefonte, PA, USA). The reagents of trace metal analysis grade [i.e., H_2_O_2_ (30% *v*/*v*) and HNO_3_ (65% *v*/*v*)] and ultrapure water (resistivity: 10 mΩ cm) were supplied by J.T. Baker (Milan, Italy). The Folin–Ciocalteu reagent was purchased from Sigma-Aldrich (Steinheim, Germany). Stock solutions of Na, Mg, K, Fe, Cu, Mn, Zn, Se, Ni, Cr, Al, As, Cd, and Pb (1000 mg/L in 2% HNO_3_, each) were provided by Fluka (Milan, Italy). Depending on the targeted analyte, the internal standards employed for the normalization of the calibration procedure were: tetradecane (99% purity, Merck Life Science S.r.l., Milan, Italy), α-cholestanol (TraceCERT^®^ grade, Supelco, Bellefonte, PA, USA), and rhenium (Re, 1000 µg/mL in 5% HNO_3_, LGC Standards, Teddington, UK).

### 2.4. Physicochemical Properties

An MB45 halogen moisture analyzer (Ohaus Corporation, Parsippany, NJ, USA) was used to estimate the moisture content of the oils. For this analysis, 2 g of each oil sample was heated to 101 °C for one hour. The moisture content was then expressed as a percentage. The refractive index is defined as the sine ratio of the incidence angle and the light ray refraction of a given wavelength passing from the air through the oil, maintained at a constant temperature. The refractive indices of oil samples were determined in accordance with the standard NF T60–212 method [33]. The measurement was conducted using an Abbe refractometer (Model RMT, Optech, Milano, Italy), equipped with a thermometer, with a scale ranging from 20 to 80 °C. The oil sample was placed on the refractometer slide and the instrument’s knobs were adjusted, while observing the boundary line through the eyepiece. Once the boundary line between the light and dark fields was sharp and clear, the refractive index was recorded using the instrument’s scale. The specific weight of the oil was determined using a density bottle, according to the methods indicated by AOAC [34]. The weight of an empty 5 mL density bottle was recorded, followed by the weight of a density bottle filled with water. An equivalent quantity of oil replaced the water in the same bottle and was weighed. The oil density was calculated using the expression:

$$Specific\;gravity=\frac{w_{1}-w_{0}}{w_{2}-w_{0}}$$

where *w*_0_; is the weight of the empty density bottle, *w*_1_ is the weight of the empty density bottle filled with water (g), and *w*_2_ is the weight of the density bottle filled with oil (g).

The saponification number is defined as the amount, in milligrams, of potassium hydroxide (KOH) required to saponify one gram of fat. This parameter was evaluated according to the protocol described by AOAC [34]. Around 2 g of each oil sample was placed in a 250 mL decantation flask and 25 mL of 0.5 N methanolic KOH was added. The flask was connected to a reflux condenser and heated for one hour to ensure the complete saponification of the fat. While the solution was still hot, it was titrated with 0.5 N HCl, using phenolphthalein as the indicator, until a colorless end point was reached. A blank titration was carried out simultaneously. The saponification number was calculated according to the following equation:

$$Saponification\;index\;(mgKOH/g)=\frac{(V_{0}-V_{1})\times N\times {KOH}_{MW}}{w}$$

where *V*_0_ is the titrant volume (ml of HCL) used for the blank titration, *V*_1_ is the titrant volume (ml of HCl) used for the sample, *N* is the HCl normality (0.5), *KOH_MW_* is the molecular weight of KOH (56.1 g/mol), and *w* is the weight of the oil sample (g). For the determination of the free acidity, the procedure described by Costa et al. was followed [35]. A solution of 90 mL ethyl alcohol/diethyl ether (1:2, *v*/*v*) was mixed with a few drops of 1% phenolphthalein and then neutralized with a KOH solution (0.1 N). The mixture was then added to a 5 g oil sample and titrated with KOH (0.1 N), until a color change was observed. The acidity was calculated using the following equation and expressed as the % of oleic acid:

$$Oleic\;acid\;(\%)=\frac{N\times V\times {AO}_{MW}}{w\times 10}$$

where *V* is the volume of the titrant (ml of KOH), *N* is the normality of KOH (0.1), *OA_MW_* is the molecular weight of the oleic acid (282 g/mol), and *w* is the weight of the oil sample (g). The peroxide value was determined following the protocol described by Costa et al. [35]. Around 25 mL of a glacial acetic acid/chloroform solution (3:2, *v*/*v*) was mixed with 500 µL of a saturated KI solution. After vigorous shaking, the solution was left in the dark for approximately 5 min. Next, 75 mL of distilled water and starch indicator were added to the mixture. The solution was then titrated with a Na_2_S_2_O_3_ solution (0.01 N), until a color change was observed. The peroxide value, expressed as milliequivalents of reactive oxygen per kilogram of oil sample (mEqO_2_/kg), was calculated using the following equation:

$$Peroxide\;value({mEqO}_{2}/kg)=\frac{V\times N\times 1000}{w}$$

where *V* is the titrant volume (ml of Na_2_S_2_O_3_), *N* is the normality of the Na_2_S_2_O_3_ solution (0.01), and *w* is the weight of the oil sample (g).

### 2.5. Fatty Acid (FA) Composition

The protocol followed in this study in regard to the FA composition was previously used by Lo Turco et al. [36]. Approximately 0.1 g of oil was mixed with 2 mL of n-heptane and 0.2 mL of KOH solution in methanol for 30 s at room temperature, followed by decantation. The upper layer containing fatty acid methyl esters (FAMEs) was then sampled and subjected to analysis via gas chromatography (GC), using a Dani Master GC1000 chromatograph equipped with a split/splitless injector and flame ionization detector (FID) (Dani Instrument, Milan, Italy). Chromatographic separation was achieved using a SLB-IL100 capillary column, 60 m in length, with a diameter of 0.25 mm, and a film thickness of 0.20 µm (Supelco, Sigma Aldrich, Burlington, MA, USA). The operating conditions included a column oven temperature ranging from 165 to 210 °C, increasing at a rate of 2 °C/min, and maintained at this temperature for 10 min. The injector and detector temperatures were set at 250 °C, with helium gas used at a constant linear velocity of 30 cm/s. The injection volume was 1 µL and a fractionation ratio of 1:100 was applied. Data acquisition and processing were carried out using Clarity v. 4.0.2 chromatography software. The FAMEs of nutritional interest were identified by direct comparison with the retention times of the reference compounds and quantified by the percentage peak area method (i.e., the quantity of every target FA is expressed as a percentage of the relative peak area, in relation to the total chromatogram area).

### 2.6. Tocopherol Analysis

The determination of the α, γ, and δ tocopherols occurred following the procedure proposed by Amar et al. [37]. For the sample preparation, 100 µL of the oil sample was diluted in 1 mL of n-hexane and filtered through a 0.20 µm PTFE syringe filter. Analysis was then conducted using a high-performance liquid chromatography system, coupled to a fluorescence detector (HPLC-FD, Shimadzu, Milan, Italy). The chromatographic separation was performed using a LiChrosorb^®^ Si60 column (250 mm × 4.6 mm L.D., 5 µm particle size, Merck), protected by a LiChroCART 4–4 guard column with the same stationary phase (Merck), and exploiting a mobile phase composed of n-hexane/ethyl acetate (90:10 *v*/*v*) under isocratic conditions. The HPLC-FD analyses were performed at 40 °C, with an injection volume of 20 µL and a flow rate of 0.8 mL/min. Data processing was carried out using the LabSolutions software (v. 5.10.153, Shimadzu). The identification of tocopherols occurred by direct comparison with the retention time of the commercial standards at excitation and emission wavelengths of 295 nm and 330 nm, while the quantitative analysis was performed using an external calibration procedure.

### 2.7. Sterol Analysis

The sterol profile of the oil samples was investigated according to the procedure suggested by EU Regulation n. 1348/2013 [38]. Every oil sample was directly added to the internal standard α-cholestanol and saponified using a KOH solution in ethanol. The obtained mixture was extracted with ethyl ether to obtain the unsaponifiable fraction. Then, the sterols were separated from the unsaponifiable matter by thin layer chromatography (TLC). For this purpose, every ethyl ether solution was loaded onto glass plates (20 × 20 cm) coated with a basic silica gel (type G), which was previously activated by heating at 110 °C for 90 min. Elution was performed in 45 min with 100 mL of *n*-hexane/ethyl ether (65:35 *v*/*v*) in a glass developing chamber (27.0 × 26.5 × 7.0 cm). The plates were then sprayed with an ethanolic solution of 2,7-dichlorofluorescein (0.2%, *w*/*v*) to highlight the bands under a UV source (366 nm). The band of sterols was scraped off the silica gel and extracted with 10 mL of hot ethyl acetate. After removing the ethyl acetate under a vacuum, the residue was derivatized by using 0.1 mL of BSTFA–TMCS (99:1, *v*/*v*) at room temperature for 30 min. Trimethylsilyl ether (TMSE) derivatives were then analyzed by GC/FID (Dani Master GC1000, Dani Instrument, Milan, Italy), using a SPB-1 capillary column (15 m × 0.20 mm ID, 0.20 µm film thickness, Supelco, Bellefonte, PA, USA). The oven temperature program ranged from 240 °C (5 min hold) to 290 °C (5 min hold) at 2 °C/min. The injector and detector temperatures were, respectively, 280 °C and 290 °C. Helium was used at a (constant) linear velocity of 30 cm/s, while the injection volume was 1 µL, with a split ratio of 1:50. Individual phytosterols were identified based on the retention time of the commercial standard and quantified by the internal standard method.

### 2.8. Squalene Analysis

Squalene was extracted from the oil samples and analyzed, as already reported by Vadalà et al. [39]. Around 0.1 g of oil previously added to the internal standard tetradecane was passed through a Supelco Discovery DSC-Si silica solid phase extraction (SPE) cartridge and eluted with n-hexane. For the analysis, a gas chromatography system (GC-2010, Shimadzu, Milan, Italy), coupled to a single quadrupole mass spectrometer (QP-2010 Plus, Shimadzu, Milan, Italy), was employed. The chromatographic separations were performed on an SPB-5 MS capillary column (30 m × 0.25 mm inner diameter × 0.25 µm film thickness, Supelco, Bellefonte, PA, USA). The oven temperature program ranged from 80 °C (held for 1 min) to 140 °C at 20 °C/min and, finally, to 290 °C (held for 2 min) at 5 °C/min. The injection port temperature was set at 250 °C and the injection volume was 1 µL, with a fractionation ratio of 1:10. The MS conditions were as follows: EI source temperature 230 °C; ionization energy and emission current 70 eV and 250 µA, respectively; interface temperature 290 °C. Identification was performed through a full scan (mass range: 40–400 *m*/*z*), comparing the retention time and mass spectrum with those of the commercial standard, while quantification was performed through selected ion monitoring (SIM) of four characteristic mass fragments (121, 137, 161, and 175 *m*/*z*). Hence, the quantity of the compound was calculated considering the relative base peak ions and exploiting the normalization in accordance with the internal standard.

### 2.9. Total Polyphenol Assay

The total polyphenolic content of the oil samples was evaluated by following the procedure reported by Albergamo et al. [40]. First, 2 mL of the oil sample was mixed with 2 mL of a methanol/acetic acid/water solution (50:8:42, *v*/*v*/*v*). Then, the mixture was stirred for 20 min and centrifuged at 6630 g for 5 min. The obtained supernatant was filtered consecutively through 0.45 μm and 0.20 μm polytetrafluoroethylene filters. An aliquot of the extracted sample (1 mL) was added to 5 mL of Folin–Ciocalteu reagent and 10 mL of Na_2_CO_3_ solution (20%) in a 100 mL flask and distilled water was added up to the fill mark. After incubating in the dark for 120 min, the solution was read at 760 nm with a UV/visible spectrophotometer (UV-2401 PC, Shimadzu, Kyoto, Japan). For the determination of the total polyphenol content of the oil samples, gallic acid was used as the analytical standard for building up a six point calibration curve. Hence, the results were expressed as mg of gallic acid equivalent per kg of oil (mg GAE/kg).

### 2.10. Inorganic Elements

The oil samples were digested by following the protocol by Nava et al. [41] and using an Ethos I microwave digestion system (Milestone), equipped with temperature and pressure sensors and PTFE (polytetrafluoroethylene) vessels capable of withstanding pressures of up to 110 bar. Initially, 0.3 g of each sample was accurately weighed in the PTFE vessels, 1 mL of the internal standard Re at a known concentration was added and mineralized with 7 mL of HNO_3_ and 1 mL of H_2_O_2_. After cooling, the samples were diluted to 25 mL with distilled water and filtered using a 0.45 μm syringe filter to remove insoluble particles. Elemental analyses were performed using a quadrupole iCAP Q ICP-MS (Thermo Scientific, Waltham, MA, USA), equipped with an ASX-520 autosampler. Before the analysis, the ICP-MS method was analytically optimized and validated, as already reported by Nava et al. [41]. The operating parameters were as follows: incident RF power equal to 1550 W, and plasma (Ar), auxiliary (Ar), and carrier (Ar) gases at flow rates of 14 L/min, 0.8 L/min, and 1.10 L/min, respectively. The instrument was operated in He collision mode (4.7 mL/min) and with the spray chamber set at +2.7 °C. The injection volume and sample introduction rate were 200 µL and 0.93 mL/min, respectively. The spectra were acquired in full scan mode (a dwell time of 0.5 or 0.01 s/point, depending on the analyte). The instrumental control and data acquisition were performed by Thermo Scientific’s Qtegra™ Intelligent Scientific Data System software (https://www.thermofisher.cn/order/catalog/product/IQLAAEGABSFAOVMBCZ (accessed on 26 August 2024). Thermo Fisher Scientific Inc., Waltham, MA, USA). For the quantification, an external calibration procedure based on the construction of seven-point calibration curves and combined with internal standard normalization was used.

### 2.11. Statistical Analysis

In this study, a total of *n* = 3 wild oil samples and *n* = 3 domesticated oil samples were considered, so that the experimental data from a given type of oil were expressed as the mean ± standard deviation of *n* = 3 samples, where every sample was analyzed in triplicate. The data were analyzed statistically using R Studio v. 4.2.1. After performing a Shapiro–Wilk test to verify the normal distribution of the experimental data, each parameter was statistically analyzed in both sets of oil samples using a two-tailed Student’s *t*-test for unpaired data, with a statistical significance set at *p* ≤ 0.05 for all statistical analyses.

## 3. Results and Discussion

### 3.1. Yield and Physicochemical Properties

In the first instance, the evaluation of the yield, along with the physicochemical characteristics of an oil are relevant for determining the profitability of the plant and the commercial quality of the derived oil, as well. Table 2 presents the basic analysis parameters of *B. aegyptiaca* oils.

The oil content from the cultivated species was significantly higher than that of its wild counterpart (42.36% vs. 36.17%, *p* < 0.05). Khadra et al. analyzed the same oil from the Algerian provinces Adrar, Beni-Abbes, Tamnrasset, and Saoura and highlighted a much lower extraction yield (23.33% and 29.47%) [19,25]. Overall, the literature revealed that the oil potential of *B. aegyptiaca* can vary considerably (14.08–69%) in relation to the growth scenario and the extraction method (e.g., mechanical pressing or solvent extraction) [18,20,21,42,43].

In our study, significant differences were also found in regard to the saponification number, free acidity, and the peroxide value. The saponification index is indicative of the average chain length (or molecular weight) of the FAs present in an oil. A low saponification index reflects the presence of long-chain FAs in the triglycerides of an oil, while a high index indicates triacylglycerols with shorter FAs [44]. The saponification value of the domesticated desert date oil was higher than that recorded for the wild oil (162.69 mg KOH/g vs. 157.48 mg KOH/g, *p* < 0.05), as it presumably had a higher amount of low molecular weight FAs. However, the results are below the threshold of 184–196 mg KOH/g set by the Codex Alimentarius for virgin olive oil [45]. A literature review showed that both the saponification indices were comparable (162.4 mg KOH/g [46]) or even lower than those of the same oil from wild fruits collected in different geopedoclimatic conditions (range: 168.60–232 mg KOH/g) [19,21,22,24,42,43,47,48], and for common edible oils, such as peanut (188–196 mg KOH/g), corn (187–196 mg KOH/g) [49], canola (175.1–188.8 mg KOH/g), olive (179.6–186.1 mg KOH/g), and sunflower (178.1–81.5 mg KOH/g) oils [50]. The level of free acidity is a good indicator of the degradation of triglycerides in an oil and the subsequent liberation of FAs. In the case of desert date oil, domestic and wild oils showed, respectively, a free acidity equal to 0.16% and 0.34% (*p* < 0.05), both of which are lower than the recommended value of 2% set for edible fats and oils not covered by individual standards by the Codex Alimentarius Commission [51]. Khadra et al. highlighted slightly higher, but still acceptable, free acidities in *B. aegyptiaca* oils from different provinces in south Algeria (0.45–0.50%) [19]. Similarly, a satisfactory range was reported in the literature for the same type of oil (0.14–0.82%) [24,43,47,48,52]. The peroxide value is a useful criterion for evaluating the initial stages of oxidative deterioration of an oil. Non-significantly different peroxide values of 3.60 mEqO_2_/kg and 4.60 mEqO_2_/kg were recorded for domestic and wild oils (*p* < 0.05). Such values are well below the threshold of 10 mEqO_2_/kg set by the Codex Alimentarius Commission [51]. In the literature, the wild desert date oil from Algeria had similar peroxide values (3.73–4.13 mEqO_2_/kg) [19], while the oil produced in other geographic and climatic contexts showed noticeable variability, as similar (2.95–4.00 mEqO_2_/kg) [22,24,43,46,48] and higher (6.00–13.34 mEqO_2_/kg) [42,47,52] peroxide levels were reported. Overall, the low saponification index, free acidity level, and peroxide value of *B. aegyptiaca* oil underline its suitability to be employed for food purposes and to be stored for a long time, due to its low oxidative and lipolytic activities. However, the statistical analysis pointed out that, for these parameters, the domesticated oil has slightly better quality than its wild relative. This could be related to the agronomic management that improved the yield and physicochemical quality of the seed oil from the experimental *B. aegyptiaca* trees.

The experimental cultivation of *B. aegyptiaca* did not have a considerable effect on the other physicochemical properties of the domesticated oil with respect to the wild oil. Similar specific gravity values (0.92 and 0.93, *p* > 0.05), lower than water (1.00), and low water content (0.25% and 0.27%, *p* > 0.05), confirmed the good stability and shelf life of the oils. Generally, water in oils is low (0.005–0.3%), as higher moisture and, consequently, specific gravity values, may damage the oil over time by promoting hydrolytic reactions [53]. According to a report by the Codex Alimentarius Commission [54], the moisture in virgin olive oil should not exceed 0.2%. In our study, the slightly higher water content in desert date oils could be related to the lack of a drying step of kernels, before oil extraction. However, our results are in line with the literature, which reported specific gravities of wild desert date oil from different African areas equal to 0.90–0.93 [18,24,42,46,47] and moisture content amounting to 0.15–0.50% [18,19,42]. Moreover, the specific gravity values were in line with those of the most common edible oils, including soybean, palm, and olive oil (0.91–0.92) [55]. The refractive index is typically used to check for the adulteration of seed oil with other vegetables oils [55]. The refractive index of both *B. aegyptiaca* oils was equal to 1.471 and 1.470 (*p* > 0.05), thus being within the ranges of the Codex Alimentarius for virgin olive oil (1.4677–1.4707) [45] and for named vegetable oils (1.448–1.477) [56]. The obtained values agree with the refractive indices of wild desert date oils from southern Algeria (1.472–1.473) [19] and from different African sites (1.41–1.47) [42,43,44,46,52], thus confirming the purity of the investigated *B. aegyptiaca* oils. Moreover, the refractive indices are comparable to those of common edible oils, such as cottonseed (1.470–1.473), sunflower (1.461–1.468), palm (1.453–1.458), and olive (1.4703) oil [57,58].

### 3.2. FA Composition

The FA composition of *B. aegyptiaca* oils is reported in Table 3. The total monounsaturated FAs (MUFAs) and polyunsaturated FAs (PUFAs), respectively, accounted for 37.70% and 33.88% in the domesticated oil and 38.95% and 33.65% in the wild oil, while saturated FAs (SFAs) constituted 28.70% and 27.40% in the domesticated and wild oil. Both types of oil showed predominant FAs, such as oleic acid (C18:1n-9, 36.03–37.40%, *p* > 0.05), linoleic acid (C18:2n-6, 33.80–33.59%, *p* > 0.05), palmitic acid (C16:0, 15.64–15.38%, *p* > 0.05), and stearic acid (C18:0, 12.12–11.41%, *p* > 0.05). Interestingly, oleic and linoleic acids represented, respectively, almost the totality of the MUFAs (95.57–96.02%) and PUFAs (99.76–99.82%) in both oils. Additionally, both palmitic and stearic acids represented 97.67% and 97.77% of the total SFAs, respectively, in the cultivated and wild oils. As a result, other FAs, such as lauric (C12:0), myristic (C14:0), arachidic (C20:0), and linolenic (C18:3 n-6) acids, were identified at very low proportions, not exceeding 0.36%. No significant differences were detected between the two types of oil in terms of the percentage of most fatty acids (*p* > 0.05). The only exceptions constituted shorter SFAs (i.e., lauric and myristic acids) and eicosenoic acid (C20:1n-9), which were significantly more abundant in the domesticated oil rather than its wild counterpart. Interestingly, the higher content of shorter SFAs in the oil from cultivated trees could explain its slightly higher saponification index compared to the wild oil. In general, it can be stated that the cultivation practice involving *B. aegyptiaca* did not have a significant impact on the FA composition of the derived oil and the variations discussed in terms of the minor FAs could be related to the natural variability in the seeds. Consequently, the FA composition of wild and domesticated oils had a similar nutritional value. This is in line with previous research on the effects of domestication on the nutritional value of different legume seeds, whose FA composition did not show consistent differences between wild and domesticated plants [59]. Additionally, high levels of beneficial oleic and linoleic acids, along with a stable proportion of SFAs, would enhance the nutritional value of the oil.

The literature points out a certain variability in wild desert date oil with respect to the main FAs, probably due to the different geopedoclimatic conditions in which *B. aegyptiaca* grew, such as in southern Israel [18], India [20], Senegal [21], Burkina Faso [22], Mauritania and Morocco [23], Egypt [24], Nigeria [46], and Sudan [47], as well as the different oil processing methods. In these previous studies, palmitic and stearic acids oscillated, respectively, between 11.01–19.13% and 2.54–13.64%, while oleic and linoleic acids varied between 22.18–58.05% and 15.48–47.84%. According to Dubois et al., *B. aegyptiaca* oil would fall within the group of oils characterized by an FA profile containing less than 60% MUFAs, and with a lower, but significant, proportion of linoleic acid. This group includes rice bran (SFAs: 21.3%, MUFAs: 42.4%; PUFAs: 35.9%), oats (SFAs: 19.8%; MUFAs: 39.8%; PUFAs: 38.9%), ratanjyot (SFAs: 22.2%; MUFAs: 40.1%; PUFAs: 36.1%), and argan (SFAs: 17.8%; MUFAs: 45.9%; PUFAs: 35.8%) oils [60].

### 3.3. Tocopherols

The tocopherol composition is an important parameter for describing the antioxidant potential of a vegetable oil, with important implications not only for the shelf life and quality of the oil, but also for the consumer’s health [36]. In fact, these lyophilic antioxidants play pivotal roles in anti-inflammatory processes [61] and their deficiency results in a range of disorders, including neuromuscular problems [62] and cardiovascular diseases [63]. Table 4 shows that the total tocopherol content of domesticated oil is significantly lower than wild oil (84.98 mg/kg vs. 97.64 mg/kg, *p* < 0.05), due to lower amounts of each analyzed isomer. The most abundant α-tocopherol was equal to 67.41 mg/kg and 73.65 mg/kg, respectively, in domesticated and wild oil; although no statistically significant difference was revealed by the *t*-test (*p* > 0.05). As follows, γ-tocopherol (10.82 mg/kg vs. 15.76 mg/kg, *p* < 0.05) and δ-tocopherol (6.74 mg/kg vs. 8.23 mg/kg, *p* < 0.05) significantly differed between the two types of oil.

The results from this study suggest that the domestication process could be responsible for a decrease in the tocopherols in the seed oil. Accordingly, Turkish carob seed oil from wild pods displayed a higher content of α-, γ-, and δ-tocopherols, with respect to the cultivated tree [64]. Edible seeds from domesticated Fabaceae oil-source plants (e.g., peanut and lupin) had a lower total tocopherol content than their wild relatives [59]. Similarly, Tunisian olive oil from different wild trees had comparable or even higher contents of α-tocopherol than the renowned Chemlali cultivar [65].

However, both domesticated and wild oils reported levels of tocopherols much lower than those described in the literature for *B. aegyptiaca*; although, the isomers maintained the same quantitative distribution. In fact, Khadra et al. [19] pointed out an α-tocopherol content of 580.80 mg/kg for the oil from the Algerian province of Saoura, obtained using a mechanical press. The oil from wild seeds in Sudan extracted by a solvent revealed total tocopherols equal to 398.5–422.0 mg/kg, with an α-tocopherol content varying between 212.0–231.0 mg/kg [47]. Cold-pressed desert date oils from Morocco, Sudan, and Mauritania had an α- and γ-tocopherol content ranging between 324–607 mg/kg and 120–226 mg/kg, respectively, while the δ-tocopherol content was in the range of 3–14 mg/kg [23]. The total tocopherols in the solvent extracted oil from Senegal amounted to 512.4 mg/kg, with an α-tocopherol content of 343.4 mg/kg [21]. Consequently, the desert date oils from this study were characterized by lower amounts of tocopherols than common vegetable oils. For example, Gliszczyńska-Świgło et al. focused on oils from the polish market and reported total tocopherols varying from 121 mg/kg (grapeseed) to 829 mg/kg (corn) in relation to the type of processing (i.e., cold pressing or refining) [66]. Khan et al. highlighted a total tocopherol content of 405.70 mg/kg, 233.20 mg/kg, and 292.70 mg/kg, respectively, for soybean, sunflower, and mustard oils from local markets in Bangladesh [67]. However, similarities in the content of single tocopherols, namely α- and δ-isomers, can be highlighted. In fact, virgin argan oil was marked by an α-tocopherol content between 30 mg/kg and 70 mg/kg, in relation to the extraction system [68]. A similar α-tocopherol content was also determined in a Tunisian cold-pressed apricot kernel oil (59.32 mg/kg) [39], while the amount of δ-tocopherol was comparable to that of cold-pressed rapeseed oil (9.3 mg/kg) and sunflower oil, both cold pressed and refined (10.1 mg/kg and 8.8 mg/kg) [66].

### 3.4. Sterols

Phytosterols are considered to be healthy components of vegetable oils, due to their potential to reduce blood cholesterol and prevent cardiovascular diseases [69]. In terms of the sterol profile, β-sitosterol is typically the most abundant sterol in an oil and it is well known for its multiple biological activities, including anxiolytic and sedative effects, and analgesic, antimicrobial, anticancer, hepatoprotective, antioxidant, and antidiabetic properties [70]. The composition of single sterols and the total sterol content were determined in both domestic and wild oils from the desert date, and the results are presented in Table 5.

Twelve different phytosterols were detected in *B. aegyptiaca* oils. The domesticated oil had a lower, but not significantly different, total sterol content than the wild oil (respectively, 935.23 mg/kg and 955.92 mg/kg, *p* > 0.05). Similarly, single sterols were generally lower in the domesticated than the wild oil; although, in most cases, non-significant differences were recorded. As expected, β-sitosterol was the main sterol in cultivated and wild oils (respectively, 535.44 mg/kg and 539.85 mg/kg, *p* > 0.05), followed by stigmasterol (respectively, 268.24 mg/kg and 276.02 mg/kg, *p* < 0.05), Δ5-avenasterol (respectively, 88.64 mg/kg and 86.71 mg/kg, *p* > 0.05), and campesterol (respectively, 21.79 mg/kg and 26.63 mg/kg, *p* < 0.05). According to the obtained data, the domestication process could somewhat negatively impact the sterol composition, especially with respect to the content of brassicasterol, campesterol, and stigmasterol. However, this is in contrast with the study conducted by Matthaus and Özcan [64], which found an improved sterol composition in carob oil following cultivation. Hence, more research should be devoted to the influence of cultivation on the sterol profile of oil-source plants.

A literature review confirmed that β-sitosterol, stigmasterol, and campesterol were the most abundant sterols in desert date oil [21,23,24,46]. In particular, El Harkaoui et al. highlighted a remarkable variability in the total sterol content of cold-pressed oils from Sudan, Mauritania and Morocco (871–2218 mg/kg), with β-sitosterol varying between 570 mg/kg and 1295 mg/kg [23]. Dhiedhiou et al. revealed higher total sterols in wild *B. aegyptiaca* oil produced in Senegal (2110 mg/kg), due to greater levels of β-sitosterol (750 mg/kg), stigmasterol (600 mg/kg), and Δ5-avenasterol (200 mg/kg) [21].

While recognizing the variability in phytosterol composition, even within the same plant source, due to differences in the agronomic and processing conditions, the geopedoclimatic context or variety, a bibliographic comparison of *B. aegyptiaca* oil with other edible plant oils pointed out similarities in the content of β-sitosterol with peanut (472 mg/kg), borage (593 mg/kg), and coconut (450 mg/kg) oils, campesterol with olive oil (22–23 mg/kg), and stigmasterol with soybean (307–458 mg/kg), sesame (241–269 mg/kg), wheat germ (210 mg/kg), and sunflower (280 mg/kg) oils [71,72].

### 3.5. Squalene

Squalene is a sesquiterpene present in the unsaponifiable fraction of common and specialty edible oils at variable levels. Notoriously, it provides several beneficial effects to the consumer’s health, such as a reduction in cholesterol and triglycerides in blood and protection from a variety of cancers [73]. As shown in Table 6, a squalene content equal to 13.43 mg/kg for domestic oil and 15.34 mg/kg for its wild relative were recorded. Although the mean squalene content was slightly higher in the wild oil, the statistical analysis did not report a significant difference between the two types of oil (*p* > 0.05). This could be indicative of the fact that the domestication process does not significantly alter the content of this bioactive in oil.

To the best of the authors’ knowledge, only Ahmed et al. [24] and Muhammad et al. [52] have investigated the squalene content in *B. aegyptiaca* oil from Egypt (0.9%) and Nigeria (13.50%), respectively, and a comparison is not possible because they expressed the results as a percentage with respect to the total chromatogram area. However, according to the obtained data, desert date oil is marked by squalene levels similar to that of cold-pressed macadamia and coconut oils (22.90 mg/kg and 20.37 mg/kg) [74] and certain varieties of apricot kernel oil (12–16 mg/kg) [75]. However, it should always be borne in mind that the content of this antioxidant may vary considerably in relation to genetic, environmental, and agronomic factors [76,77].

### 3.6. Total Polyphenols

The synergism between polyphenols and tocopherols notoriously improves the antioxidant potential of vegetable oils, thus positively affecting their shelf life [78]. Moreover, due to their predominant antioxidant and anti-inflammatory properties, polyphenols are increasingly sought after health compounds in consumers’ diets. However, the biological activity of phenolics notoriously depends on the compound family and content, which, in turn, is related to the plant source and its genotype, the context of the growth, and, not least, the oil extraction method [79]. The levels of total polyphenols detected in *B. aegyptiaca* oil are shown in Table 6. The domestic oil had a lower total polyphenol content than the wild oil, with a statistically significant difference (60.52 mg GAE/kg vs. 67.89 mg GAE/kg, *p* > 0.05).

Dabbou et al. [65] evaluated the effect of the domestication process on the chemical composition of virgin olive oils from Tunisian oleasters and Chemlali and Neb Jamel cultivars. They found a significantly greater phenol content in the oil from wild trees (340.17–832.95 mg GAE/kg) than the cultivated relatives (323.76–825.41 mg GAE/kg). Chacon-Fuentes et al. [80], characterized the flavonoid profile of wild and cultivated Chilean guava berry (*Ugni molinae* Turcz.) and single flavonoids were significantly higher in wild plants than in their cultivated counterpart. This may suggest that a reduction in polyphenols following domestication of *B. aegyptiaca* could compromise the oxidative stability and the nutritional value of the derived oil, but could also decrease the plant’s natural defenses, thus making it more susceptible to potential pathogens [80].

In past studies, the total polyphenols in desert date oil have received little attention. In fact, Khadra et al. [19] revealed total polyphenols equal to 24 mg GAE/kg of wild oil from Algeria, while Ahmed et al. [24] obtained a content of 120 mg of caffeic acid equivalents per kg of Egyptian *B. aegyptiaca* oil. Despite the discrepancy between the results produced so far, desert date oil can be defined as a good source of polyphenols. A recent study evaluating the total phenols in different commercial edible oils using the Folin–Ciocalteu assay, for example, confirmed the highest polyphenol content in olive and sesame oils (331.2 mg GAE/kg and 211.3 mg GAE/kg). As follows, canola oil had total phenols comparable to our desert date oil (57.7 mg GAE/kg), while wheat germ, rice bran, linseed, walnut, peanut, and corn oils had lower values (range 25.2–10.3 mg GAE/kg). Furthermore, soybean, sunflower, camelina, and palm oils had even lower total phenolic contents, which were less than 10.0 mg GAE/kg [81].

### 3.7. Inorganic Elements

Similar to the other components in plant oil, the profile of inorganic elements can be affected by extrinsic and intrinsic variables. First and foremost, the geographical origin and growth context define the pool of inorganic elements in the soil, which, in turn, are inevitably adsorbed by the plant and, and, to follow, the processing and storage practices, can cause the oil to be contaminated with various elements, including heavy metals [82]. Similar to other minor compounds, the profile of inorganic elements can be related to the nutritional value, as well as the product quality, of an edible oil. For example, elements, such as Cu, Fe, Mn, and Se, are essential in consumer nutrition as they function as catalysts in enzyme systems and actively participate in many cellular reactions of the metabolism [83]. Additionally, Cu, Fe, and Mn act as pro-oxidants, while Se is a precious antioxidant, and their balance may affect the oxidative stability of an oil [84]. Table 7 shows the profile of the inorganic elements in the domesticated and wild *B. aegyptiaca* oils.

The major elements were quantitatively determined in both types of oil in the order Na > Ca > K > Mg, with no statistically significant differences, excluding Ca. Indeed, domestic and wild oils were characterized by similar levels of Na (20.52 mg/kg vs. 21.34 mg/kg, *p* > 0.05), K (13.39 mg/kg vs. 12.42 mg/kg, *p* > 0.05), and Mg (8.47 mg/kg vs. 9.32 mg/kg, *p* > 0.05), while Ca was significantly more abundant in the domesticated oil than the wild one (16.75 mg/kg vs. 11.68 mg/kg, *p* < 0.05). On the other hand, trace elements showed a greater variability between the two types of oils, as demonstrated by Student’s *t*-test. In this respect, Fe had a higher concentration in the wild oil than its domestic relative (7.14 mg/kg vs. 8.75 mg/kg, *p* < 0.05). To follow, other elements, such as Zn, Mn, Cr, Cu, and Ni, varied between 0.28 mg/kg and 0.04 mg/kg, with Mn being significantly more abundant in the wild oil (*p* < 0.05) and Cr in the domesticated one (*p* < 0.05). Se, a powerful antioxidant, showed a significantly higher content in the wild desert date oil than its domesticated counterpart (0.06 mg/kg vs. 0.10 mg/kg, *p* < 0.05). Among the trace elements, Pb was the only heavy metal quantified in both the domestic and wild oils (0.02 mg/kg and 0.07 mg/kg, *p* < 0.05), while As and Cd were below the respective instrumental limits of quantification (LOQs).

Given that both cultivated and wild *B. aegyptiaca* share the same growth context (Figure 1 and Table 1) and that the agronomic management of cultivated plants did not involve any chemical inputs, it can be stated that the domestication process did not impact the element profile of the oil and that the variations discussed could be related to the natural variability of seeds. Differently from *B. aegyptiaca* seeds [85,86], to the best of the authors’ knowledge, the profile of the inorganic elements in the derived oil has barely been studied. In this respect, Zang et al. [48] revealed that the oil extracted by solvent from Nigerian fruits was characterized by much higher and not comparable contents of major and trace elements. This could be due not only to the different growth scenarios of the fruits, but also to the fact that solvent extraction is more effective than cold pressing in retaining the original profile of the inorganic elements of the seeds in the oil.

## 4. Conclusions

For the first time, a comparative study of the physicochemical and compositional qualities of the oil from cultivated and wild *B. aegyptiaca* trees was conducted. The obtained data pointed out that the experimental domestication process did not affect the physicochemical and compositional properties of the oil, in terms of the FA composition and inorganic elements and slight variations, not always statistically significant, were observed in terms of the tocopherols, sterols, squalene, and total polyphenols. Hence, the domesticated oil can be considered of the same quality as the wild oil and suitable for human consumption, as well. Clearly, these preliminary findings need to be better explored and implemented, with further research on certain aspects not addressed in this study. In fact, the impact of the domestication of indigenous fruit trees on the quality of the derived goods also deserves to be investigated in relation to other extrinsic variables, such as the selection of putative cultivars, the choice of the most appropriate agronomic practices, and postharvest processing.

Hopefully, this study will encourage the domestication of *B. aegyptiaca* as a viable strategy for enhancing the socioeconomic value of the desert date, promoting its sustainable cultivation, and contributing to food security and nutritional health in Algeria.

## Figures and Tables

**Table 1:** Geopedoclimatic characteristics of the areas selected for this study.

Site	Geographic Coordinates	Altitude	Annual Precipitation	Annual Temperature	Soil
National Forest Research Institute (NFRI) Adrar nursery	27.87718° N 0.27909° W	257 m	20.16 mm	25.3 ± 2.4 °C	Sandy clay
Matriouene	27.4800° N 1.1959° E	250 m			

**Table 2:** Physicochemical properties of oil from domesticated and wild *B. aegyptiaca*. Results are expressed as mean ± standard deviation of *n* = 3 samples per type of oil, where every sample was analyzed in triplicate.

Parameter	Oil from Domesticated Trees	Oil from Wild Trees
Yield (%)	42.36 ± 2.01 *	36.17 ± 3.50 *
Moisture content (%)	0.35 ± 0.04	0.37 ± 0.03
Refractive index	1.471 ± 0.00	1.470 ± 0.00
Specific gravity	0.92 ± 0.21	0.93 ± 0.35
Saponification number (mg KOH/g oil)	162.69 ± 1.37 *	157.48 ± 2.83 *
Free acidity (%)	0.16 ± 0.04 *	0.38 ± 0.11 *
Peroxide value (mEqO_2_/Kg)	3.46 ± 0.28 *	4.60 ± 0.49 *

* Significantly different values between domestic and wild oils (*p* < 0.05).

**Table 3:** FA composition (g/100 g of lipid extract) of the oil from domesticated and wild *B. aegyptiaca*. Data are expressed as the mean ± standard deviation of *n* = 3 samples per type of oil, where every sample was analyzed in triplicate.

FAs	Oil from Domesticated Trees	Oil from Wild Trees
C12:0	0.20 ± 0.01 *	0.02 ± 0.01 *
C14:0	0.16 ± 0.01 *	0.07 ± 0.01 *
C16:0	15.64 ± 0.71	15.38 ± 0.73
C17:0	0.12 ± 0.02	0.13 ± 0.03
C18:0	12.12 ± 0.52	11.41 ± 0.43
C20:0	0.36 ± 0.02	0.35 ± 0.02
C22:0	0.05 ± 0.01	0.03 ± 0.02
C24:0	0.04 ± 0.01	0.02 ± 0.02
*SFAs*	*28.70 ± 0.89*	*27.40 ± 1.18*
C16:1 n-9	0.05 ± 0.01	0.07 ± 0.01
C16:1 n-7	0.23 ± 0.03	0.24 ± 0.02
C17:1	0.06 ± 0.01	0.05 ± 0.01
C18:1 n-9	36.03 ± 1.16	37.40 ± 1.08
C18:1 n-7	1.19 ± 0.10	1.11 ± 0.07
C20:1 n-9	0.15 ± 0.02 *	0.09 ± 0.02 *
*MUFAs*	*37.70 ± 1.02*	*38.95 ± 1.14*
C18:2 n-6	33.80 ± 1.9	33.59 ± 1.17
C18:3 n-6	0.02 ± 0.01	0.01 ± 0.01
C18:3 n-3	0.06 ± 0.01	0.05 ± 0.01
*PUFAs*	*33.88 ± 1.9*	*33.65 ± 1.17*

* Significantly different values between domestic and wild oils (*p* < 0.05). SFAs: saturated fatty acids; MUFAs: monounsaturated fatty acids; PUFAs: polyunsaturated fatty acids.

**Table 4:** Tocopherol content (mg/kg) of the oil from domesticated and wild *B. aegyptiaca*. Data are expressed as the mean ± standard deviation of *n* = 3 samples per type of oil, where every sample was analyzed in triplicate.

Tocopherol	Oil from Domesticated Trees	Oil from Wild Trees
α-tocopherol	68.41 ± 3.96	73.65 ± 3.80
γ-tocopherol	10.82 ± 1.57 *	15.76 ± 1.83 *
δ-tocopherol	6.74 ± 0.47 *	8.23 ± 0.43 *
Total tocopherols	84.98 ± 1.79 *	97.64 ± 1.70 *

* Significantly different values between domestic and wild oils (*p* < 0.05).

**Table 5:** Sterol composition and total sterol content (mg/kg) of the oils from domesticated and wild *B. aegyptiaca.* Data are expressed as the mean ± standard deviation of *n* = 3 samples per type of oil, where every sample was analyzed in triplicate.

Sterols	Oil from Domesticated Trees	Oil from Wild Trees
Cholesterol	9.91 ± 1.60	13.15 ± 1.47
Brassicasterol	1.88 ± 0.20 *	2.52 ± 0.24 *
24-methylene cholesterol	1.80 ± 0.05	1.58 ± 0.17
Campesterol	21.79 ± 0.79 *	26.63 ± 0.82 *
Campestanol	4.24 ± 0.10	5.56 ± 0.90
Stigmasterol	268.24 ± 2.95 *	276.02 ± 3.53 *
Clerosterol	0.95 ± 0.06	0.95 ± 0.10
β-sitosterol	535.44 ± 4.45	539.85 ± 6.30
Δ5-avenasterol	88.64 ± 1.62	86.71 ± 1.45
Δ5,24-stigmastadienol	1.50 ± 0.14	1.52 ± 0.17
Δ7-stigmastenol	0.52 ± 0.03	0.72 ± 0.27
Δ7-avenasterol	0.33 ± 0.04	0.40 ± 0.12
Total sterols	935.23 ± 24.67	955.92 ± 24.4

* Significantly different values between domestic and wild oils (*p* < 0.05).

**Table 6:** Squalene (mg/kg) and total polyphenol content (mg GAE/kg) revealed in the oil from domesticated and wild *B. aegyptiaca*. Data are expressed as the mean ± standard deviation of *n* = 3 samples per type of oil, where every sample was analyzed in triplicate. The asterisk shows significantly different values between domestic and wild oils (*p* < 0.05 according to Student’s *t*-test).

	Oil from Domesticated Trees	Oil from Wild Trees
Squalene	13.43 ± 2.50	15.34 ± 1.82
Total polyphenols	60.52 ± 1.51 *	67.89 ± 2.09 *

* Significantly different values between domestic and wild oils (*p* < 0.05).

**Table 7:** Content of major and trace elements (mg/kg) in the oil from domesticated and wild *B. aegyptiaca*. Data are expressed as the mean ± standard deviation of *n* = 3 samples per type of oil, where every sample was analyzed in triplicate.

Element	Oil from Domesticated Trees	Oil from Wild Trees
Major elements		
Na	20.52 ± 0.58	21.34 ± 0.92
Mg	8.47 ± 0.43	9.32 ± 0.27
K	13.39 ± 0.44	12.42 ± 0.66
Ca	16.75 ± 0.61 *	11.68 ± 0.35 *
Trace elements		
Fe	7.14 ± 0.09 *	8.75 ± 0.22 *
Zn	0.27 ± 0.04	0.28 ± 0.06
Mn	0.16 ± 0.03 *	0.24 ± 0.04 *
Cr	0.12 ± 0.02 *	0.09 ± 0.02 *
Cu	0.07 ± 0.02	0.04 ± 0.01
Ni	0.04 ± 0.01	0.05 ± 0.01
Se	0.06 ± 0.01 *	0.10 ± 0.02 *
Pb	0.02 ± 0.00 *	0.07 ± 0.02 *
As	<LOQ	<LOQ
Cd	<LOQ	<LOQ

* Significantly different values between domestic and wild oils (*p* < 0.05). LOQ (limit of quantification) of: As = 0.010 mg/kg; Cd = 0.003 mg/kg.

## Data Availability

The original contributions presented in the study are included in the article, further inquiries can be directed to the corresponding authors.
